# When linguists talk mathematical logic

**DOI:** 10.3389/fpsyg.2014.00382

**Published:** 2014-05-14

**Authors:** David J. Lobina

**Affiliations:** Faculty of Philosophy, University of OxfordOxford, UK

**Keywords:** induction scheme, recursive functions, definition by induction, mathematical induction

Given the importance of recursion in modern linguistics, there ought to be much to commend in Watumull et al.'s (2014) attempt to clarify what recursion is (or ought to be); I have trudged this very terrain myself, using some of the same sources, and in order to make similar points (e.g., Lobina, 2011, but especially in Lobina, 2012). However, there are so many issues with Watumull et al.'s own attempt that a proper response is in order. I will here limit myself to the following: (a) the characterization of recursion these authors offer is wholly mistaken, the unavoidable result of misunderstanding, misrepresenting, and misinterpreting the relevant literature from the formal sciences; and b) as a corrective, I provide a definition of recursion that stands on much firmer ground in order to then show how it relates to Chomsky's introduction of recursive techniques into linguistics.

Watumull et al. (WEA, from now on) base their definition on a quote of Gödel (1931) regarding what they call the “primitive notion of recursion” (p. 2), deriving therefrom three criterial properties of recursion: (a) the function must specify a finite sequence (Turing computability, WEA claim); (b) this function must be defined in terms of preceding functions, that is, it must be defined by induction, which WEA associate with strong generativity (i.e., the generation of ever more complex structure); and (c) this function may in fact just reduce to the successor function (that is, mathematical induction, which WEA associate with the unboundedness of a generative procedure). Unfortunately, this characterization of recursion is mistaken in both design and detail.

To begin with, WEA don't provide the full version of Gödel's text, their ellipses omitting important material, as the full text demonstrably shows (I'll be quoting from Davis (1965), which offers a different translation from the one WEA use, but this won't affect my analysis):

A number theoretic function ϕ is said to be recursive if there exists a finite sequence of number-theoretic functions ϕ_1_, ϕ_2_, …, ϕ_*n*_ which ends with ϕ and has the property that each function ϕ_*k*_ of the sequence either is defined recursively from two of the preceding functions, or results [footnote not included] from one of the preceding functions by substitution, or, finally, is a constant or the successor function *x* + 1 (pp. 14–5; underline in the original).

As is clear from the full quote, what Gödel is doing here is defining a specific class of functions; he called these functions the recursive class in that text, but these are now known as the *primitive recursive functions* (Davis, 1965, p. 4). What he is absolutely not doing is defining recursion *per se*. Moreover, Gödel's definition is not actually a combination of properties subsuming any such prior concept at all, as WEA would have us believe (and they do so by omitting substitution and the constant function from the text). That is clearly *not* what the quote states. Here Gödel is merely saying that if we have a list of functions (and there is no indication that this list is computed by a Turing Machine, a notion that was unavailable to Gödel in 1931 anyway), any one function from this list will be defined as recursive if it

- is defined by induction from previous functions, OR
- is substituted by some of them, OR
- is the constant function, OR
- is the successor function.

Gödel's quote is in fact a pretty standard definition of the primitive recursive class (cf. the five recursive functions outlined in Kleene 1952, pp. 220–223). There are other types of recursive functions (such as the general and the partial), and all these functions are recursive for the same reason; they just happen to be different classes of functions because of the different input-output relations they encompass.

So what is recursion, then? As Brainerd and Landweber (1974) put it, it is useful to define functions “using some form of induction scheme…, a general scheme… which we call recursion” (p. 54). This is consonant with the original interpretation of recursion as being part of a definition by induction, as chronicled by Soare (1996). Also known as a recursive definition, it consists, as WEA themselves allude to (e.g., in page 2), in “defining a function by specifying each of its values in terms of previously defined values” (Cutland, 1980, p. 32). WEA don't actually provide an example of what a definition by induction involves, and it is important to do so. A standard example is that of the factorial class (*n*! = *n* × *n* − 1 × *n* − 2 … × 2 × 1, where *n* is a natural number), which can be recursively defined in the two-part system so typical of such definitions: (a) if *n* = 1, *n*! = 1 (base case), (b) if *n* > 1, *n*! = *n* × (*n* − 1)! (recursive step). Note that the recursive step involves another invocation of the factorial function, and it is precisely the self-referential (or self-call) property of such definitions that makes a function recursive (Tomalin, 2006, p. 61).

So what's wrong with WEA's characterization, then? The first property WEA list can be swiftly dealt with: there's no reason to equate the finite sequence of functions Gödel mentions in his 1931 paper with Turing computability; nor is there any indication in that text that such a sequence of functions is the result of a computation. As for the second property WEA outline—the identification between a definition by induction and structure generation— these are two very different things, and it is not clear why WEA make the connection at all. After all, the previously defined values that a recursive function calculates are not objects that partake in the further construction of other, more complex objects. Rather, the computation of the factorial of 4, for instance, necessitates the computation of the factorial of 3, but the latter is the value that is calculated by another function (the result of a self-call), neither internal to the factorial of 4 itself nor constitutive of its operations. This is also true of a Turing Machine (TM), which WEA describe in rather peculiar terms;^1^ all a TM does is write or erase digits on a tape according to some rules (the so-called configuration), and whilst a collection of digits could stand for many different things (as specified in the TM's look-up table at least), every operation a TM carries out is exhausted at each stage; that is, the configuration changes for each cell, making each step of a TM computation a self-contained one. Technically, therefore, there is neither structure (WEA, p. 2) nor “derivational history” (WEA, p. 4, ft. 2) being carried forward on the tape. A similar state of affairs applies to the three-way identification WEA specify regarding the third criterial property: the successor function qua mathematical induction qua the unboundedness of a generative procedure. A mathematical induction, a related and yet distinct concept to a definition by induction, is a technique employed to prove whether a given property applies to an infinite set, and proceeds as follows: first, it is shown that a given statement is true for 1; then, it is assumed that it is true for *n*, a fixed number (the inductive hypothesis); and finally, it is established that it is therefore true for *n* + 1 (the inductive step). The misidentification between mathematical induction and the successor function (recall, a primitive recursive function) could perhaps be excused on the grounds that most expositions of mathematical induction employ the successor function as a case in point (e.g., Kleene, 1952, pp. 20 et seq.), but this doesn't have to be the case: Buck (1963) provides examples of mathematical induction with many other types of data. In any case, what mathematical induction clearly isn't is unboundedness itself; as stated, mathematical induction is a technique to prove if a given statement is true of an infinite set, a different concept altogether.

I should note, in any case, that I don't dispute the importance of the three properties WEA line out for a theory of language. What I deny is that these properties are criterial of what recursion is; and more specifically, that they are associated to (or indeed identified with) the three concepts WEA selectively and mistakenly extract from Gödel (1931). Moreover, WEA provide a story that is somewhat at odds with the manner in which Chomsky introduced recursion into linguistics, including how he has always understood this notion (at least in his individual writings), and I would like to end this note with some brief comments regarding this point.

It is commonplace among linguists that the number of sentences that language users can produce and understand is unbounded, and therefore that a computational system must be postulated in order to account for this fact. Chomsky has been rather clear that generative grammar developed within ‘a particular mathematical theory, namely, recursive function theory’ for this very task (p. 101 in Piattelli-Palmarini, 1980), a choice of words that is rather illustrative. As Soare (1996) has chronicled, mathematical logic made ample use of recursive techniques in order to formalise the notion of a “computation” in the 1930s and 1940s, to the point that in subsequent decades recursion itself was taken to be synonymous with the term computation (and recursive with computable), a state of affairs Soare has called the Recursion Convention. The field has apparently moved on from this, now preferring to call itself *computability theory* instead of *recursive function theory* (Soare, 2007), perhaps on account of the fact that even though recursively-specified formalisms such as the partial recursive functions can indeed model what a computation is, many non-recursive formalisms do just as well (e.g., a Turing Machine)^2^. As I reported in Lobina (2011, p. 155, ft. 5), in fact, Chomsky has always tacitly followed the Recursion Convention, and this might help explain his insistence on the centrality of recursion.

Chomsky has been remarkably consistent in stating that the language faculty recursively enumerates syntactic objects, where a set is recursively enumerable if there is an algorithm that can list its members. Indeed, in the 1966 edition of his *Language and mind* book, re-edited in 2006, we find that a “generative grammar recursively enumerates structural description of sentences” (p. 165), a stand that is maintained in Chomsky (1981, pp. 11–13) and then re-emphasised in the 1990s with the postulation of a computational mechanism, termed *merge*, that “recursively constructs *syntactic objects* from [lexical] items… and syntactic objects already formed” (Chomsky, 1995, p. 226). To be sure, the early years of generative grammar saw the employment of Post's production systems, a more obvious recursive formalism^3^, but this aspect of the theory hasn't translated much with the advent of *merge*, given that a recent description delineates it in very general terms as a set-theoretic operation in which repeated applications over one element yield a potentially infinite set of structures, drawing an analogy between the way *merge* applies and the successor function (Chomsky, 2008). Incidentally, it bears emphasis that what used to be called a recursively enumerable set is nowadays more appropriately termed a computably enumerable set, adding to the point that Chomsky's employment of recursion may perhaps owe more to Soare's Recursion Convention than to any other single factor.

Needless to say, this narrative has little to do with Gödel's definition of the (primitive) recursive class of functions, as WEA would have it. There is in fact so much more that one could say about WEA, but this, apparently, is not the place to do so (see the acknowledgment section for directions to a longer version of this paper).

## Funding

This research was funded in part by a Beatriu de Pinós post-doctoral fellowship (ref: 2011-BP-A-00127) awarded by the Catalan Research Institute (AGAUR).

### Conflict of interest statement

The author declares that the research was conducted in the absence of any commercial or financial relationships that could be construed as a potential conflict of interest.

## Footnotes

^1^Indeed, WEA insist on talking of the outputs of a TM as being “recursed,” which can give the impression that recursion is part of its operations, but this is not the case at all.

^2^The point here is that even though these formalisms are extensionally equivalent—i.e., they converge on the same output given a common input— there are intensional differences regarding how they in fact achieve these input-output pairs (see Epstein and Carnielli, 2008 for details).

^3^As Post (1943) points out, a production system ‘naturally lends itself to the generating of sets by the method of definition by induction’ (p. 201); cf. WEA (p. 3).
